# Genomics of Atlantic Forest *Mycobacteriaceae* strains unravels a mobilome diversity with a novel integrative conjugative element and plasmids harbouring T7SS

**DOI:** 10.1099/mgen.0.000382

**Published:** 2020-06-04

**Authors:** Sergio Mascarenhas Morgado, Ana Carolina Paulo Vicente

**Affiliations:** ^1^​ Laboratory of Molecular Genetics of Microorganisms, Oswaldo Cruz Institute, Rio de Janeiro, Brazil

**Keywords:** *Mycobacterium*, *Mycobacteriaceae*, plasmid, ICE, T7SS, T4SS

## Abstract

Mobile genetic elements (MGEs) are agents of bacterial evolution and adaptation. Genome sequencing provides an unbiased approach that has revealed an abundance of MGEs in prokaryotes, mainly plasmids and integrative conjugative elements. Nevertheless, many mobilomes, particularly those from environmental bacteria, remain underexplored despite their representing a reservoir of genes that can later emerge in the clinic. Here, we explored the mobilome of the *Mycobacteriaceae* family, focusing on strains from Brazilian Atlantic Forest soil. Novel *Mycolicibacterium* and *Mycobacteroides* strains were identified, with the former ones harbouring linear and circular plasmids encoding the specialized type-VII secretion system (T7SS) and mobility-associated genes. In addition, we also identified a T4SS-mediated integrative conjugative element (ICEMyc226) encoding two T7SSs and a number of xenobiotic degrading genes. Our study uncovers the diversity of the *Mycobacteriaceae* mobilome, providing the evidence of an ICE in this bacterial family. Moreover, the presence of T7SS genes in an ICE, as well as plasmids, highlights the role of these mobile genetic elements in the dispersion of T7SS.

## Data Summary

Genomic data analysed in this work are available in GenBank and listed in Tables S1 and S2 (available in the online version of this article).

**Impact Statement**

In addition to exploring the diversity of soil bacteria isolated from a niche in the Brazilian Atlantic Forest, which is one of the main biodiversity hotspots in the world, we revealed original aspects of *Mycobacteriaceae* mobilome, as we raised evidence of unique plasmids and the first T4SS-mediated integrative conjugative element (ICE) in this family. Moreover, type-VII secretion systems are part of the accessory genome of these mobilome elements (plasmids and ICE), which contribute to the understanding of *Mycobacteriaceae* evolution. We also found evidence of horizontal gene transfer within *Mycobacteriaceae* species in this niche.

## Introduction

Bacterial evolution and adaptation are in part facilitated by the acquisition of genetic information by horizontal transfer. In this context, the mobilome represents the set of mobile genetic elements (e.g. integrons, plasmids, insertion sequences, transposons, phages, integrative conjugative elements, among others) that act intra- or inter-cellularly as vectors of gene mobility [1–4].

Conjugative elements, such as conjugative plasmids and integrative conjugative elements (ICEs), mediate their own transfer to other organisms, often carrying cargo genes. ICEs are therefore able to confer novel adaptive traits on their host cells and impact on their evolution [5–7]. These elements have a similar modular structure, clustering genes involved in the same biological function as maintenance, dissemination and regulation [3, 8]. However, while plasmids represent autonomously replicating elements, most ICEs depend on being integrated into a replicon to facilitate their inheritance [3, 9]. ICEs can be classified into two types, T4SS-mediated or AICE (actinomycete ICEs). While the former is characterized by a T4SS apparatus, relaxase and integrase, AICEs rely on a unique TraB protein (FtsK protein family) [10]. Generally, the integration of mobile elements leaves genomic marks, such as direct repeats, flanking the integrated element [11].

Mycobacteria belong to the *Mycobacteriaceae* family, which encompass ecologically, economically and clinically relevant organisms. Their members can be classified into fast- or slow-growing mycobacteria (the latter group containing the major pathogenic species), depending on the time of growth in the solid medium. Mycobacteria are flexible organisms that can inhabit a wide range of environments, including water bodies, soil, metalworking fluids, animals and humans [12, 13]. Among the mobilome elements of this family, thousands of bacteriophages have been isolated, and over 1700 have been sequenced [14]. Conversely, plasmids are supposed to be rare [15, 16]. So far, only dozens of plasmids have been characterized (https://www.ncbi.nlm.nih.gov/genome/plasmids), most of them in clinical strains [17–30], despite hundreds of species in the *Mycobacteriaceae* family. Plasmids have primarily been associated with the evolution and dissemination of a specialized secretion system, termed ESX or type-VII secretion system (T7SS), among the mycobacteria. This secretion apparatus consists of a complex of membrane and associated proteins. The T7SS is encoded by six paralogous loci (ESX-1,-2, -3,-4, -5 and -4-bis), each with variations in its genetic organization and function. While ESX-3 and ESX-4 loci are ubiquitously distributed in *Mycobacteriaceae *, ESX-2 and ESX-5 are only found in slow-growing mycobacteria, and ESX-1 distribution is variable. ESX-1 has been associated with virulence processes in slow-growing mycobacteria, while it has been reported to be involved, along with ESX-4, in horizontal gene transfer in some *Mycolicibacterium* species. ESX-5 has also been linked to virulence processes, in addition to playing a role in membrane integrity. ESX-3 is considered essential for the survival of mycobacteria, as it is involved with the uptake of iron and zinc. The function of the ESX-2 is still unknown [31–33]. Other mobilome elements, as the integrative conjugative elements, are also rare in this bacterial family. So far, AICEs, but not ICEs, have been reported in few *Mycobacterium* genomes [9, 34].

Here, we explored the mobilome of *Mycobacteriaceae *, focusing on plasmids and integrative elements based on metagenomes of soil strains from a low anthropogenic impacted region using *in silico* approaches. The analyses revealed circular and linear plasmids carrying genes resembling T7SS and T4SS. Moreover, a T4SS-mediated ICE, termed ICEMyc226, was identified and characterized in a *Mycolicibacterium* sp. strain from Atlantic Forest soil. This ICE harbours genes related to the metabolism of xenobiotics, amino acid and carbohydrate; besides encodes two ESX-systems with distinct origins (plasmid and chromosome).

## Methods

### Bacterial strains and growing conditions

The bacteria employed in this study encompassed 17 *Mycobacteriaceae* strains isolated from Atlantic Forest soils (CBMA strains) and deposited in the Bacteria Collection of Environment and Health (CBAS, Fiocruz Institute-Brazil). The CBMA strains were grown in Tryptic Soy Broth (TSB) medium up to 6 days at 23 °C.

### Public data set

Representative complete/draft genomes (*n*=47) and plasmid sequences (*n*=87) of *Mycobacteriaceae* family, and *Nocardia brasiliensis* ATCC 700358 genome (NC_018681.1) were obtained from the National Center for Biotechnology Information (NCBI) public database (July 2019). The accession numbers for these *Mycobacteriaceae* genomes are supplied in Tables S1 and S2.

### CBMA genome sequencing and assembly

In this study, we generated 17 *Mycobacteriaceae* genomes. The genomic DNA extraction was done using Purelink Genomic DNA Mini Kit (Invitrogen). The genome libraries were constructed using different single/paired-end libraries (Nextera, Truseq and Agilent) following each manufacturer’s instructions. The sequencing was performed on an Illumina Hiseq 2500 for most CBMA strains, generating reads of 100–150 bp length. The strain CBMA 360 was sequenced on 454 GS Junior, generating reads of ~500 bp length. The raw reads were filtered and trimmed (Phred quality score <20 and read length <30) using NGSQCToolkit v.2.3.3 [35] and Quake v.0.3 [36]. The genomes were *de novo* assembled with SPAdes v.3.5 or v.3.9 [37] and improved using Pilon [38].

### Sequence annotation and species phylogeny

The genomes were annotated by Prokka v1.12 [39] and submitted to orthology analysis using GET_HOMOLOGUES v3.0.5 [40] considering a minimum coverage of >=70 % and identity >=40 %. The orthologous genes that represented the core genome of the data set were concatenated and submitted to phylogenetic analysis using RAxML v8.2.12 [41] with 100 bootstrap replicates. The iTOL generated the core genome tree [42] with *Nocardia brasiliensis* as outgroup. Type-II toxin-antitoxin loci were surveyed in the CBMA mobile elements using TAfinder [43]. Sequence repeats were identified by Unipro UGENE v1.32 with a window size of 100 bp [44], and the figures generated by EasyFig v2.2.2 [45]. Comparative analyses with integrative conjugative elements used the ICEberg database [34]. Functional annotation and assign of KEGG functional categories were performed using the BlastKOALA tool [46]. The metabolic pathway map was generated using iPath3.0: interactive pathways explorer v3 [47] with the KEGG data. The genomic atlas was generated using BRIG [48]. Searches for antibiotic resistance genes were based on The Comprehensive Antibiotic Resistance Database (https://card.mcmaster.ca/) [49]. The prediction of putative horizontal gene transfer (HGT) events was performed by Alien_hunter software v1.7 [50].

### *In silico* approaches for plasmid detection

The plasmid detection in the CBMA genomes included some strategies: (i) blastn searches against NCBI plasmid database using the contigs of the CBMA genomes as queries; (ii) searches against CBMA genomes using hmm profiles of proteins associated with plasmid replication and transfer [9, 51]; (iii) topology inference, using overlapping ends of the contigs together with the paired-end reads that connect both ends of the sequences [52]; (iv) plasmidSPAdes software v.3.13, that predicts plasmids based on sequencing coverage using read sequences [53]; and (v) T7SS phylogeny, since chromosome- and plasmid-borne T7SS sequences evolve independently. Strategy (iii) made it possible to infer the circularity or linearity of the contigs.

### T7SS detection and phylogeny

The T7SS core proteins (EccA, EccB, EccC, EccD, EccE and MycP) were searched based on their domains using HMMer package v3.1b2 [54]. ESX loci were selected if at least four core genes were close to each other. The identified protein sequences were aligned with MAFFT v7.310 [55] and the low-quality alignment columns removed using GUIDANCE2 v2.02 [56]. The resulting alignments were concatenated for phylogenetic analysis. A maximum-likelihood tree was generated using PhyML v3.1 [57] with LG+I+G+F substitution model and 100 bootstrap replicates.

## Results

Here, to explore and characterize the mobilome of Atlantic Forest *Mycobacteriaceae* (CBMA strains), we sequenced their genomes and applied a set of *in silico* approaches.

### Genus inference and T7SS phylogeny

**Fig. 1.**
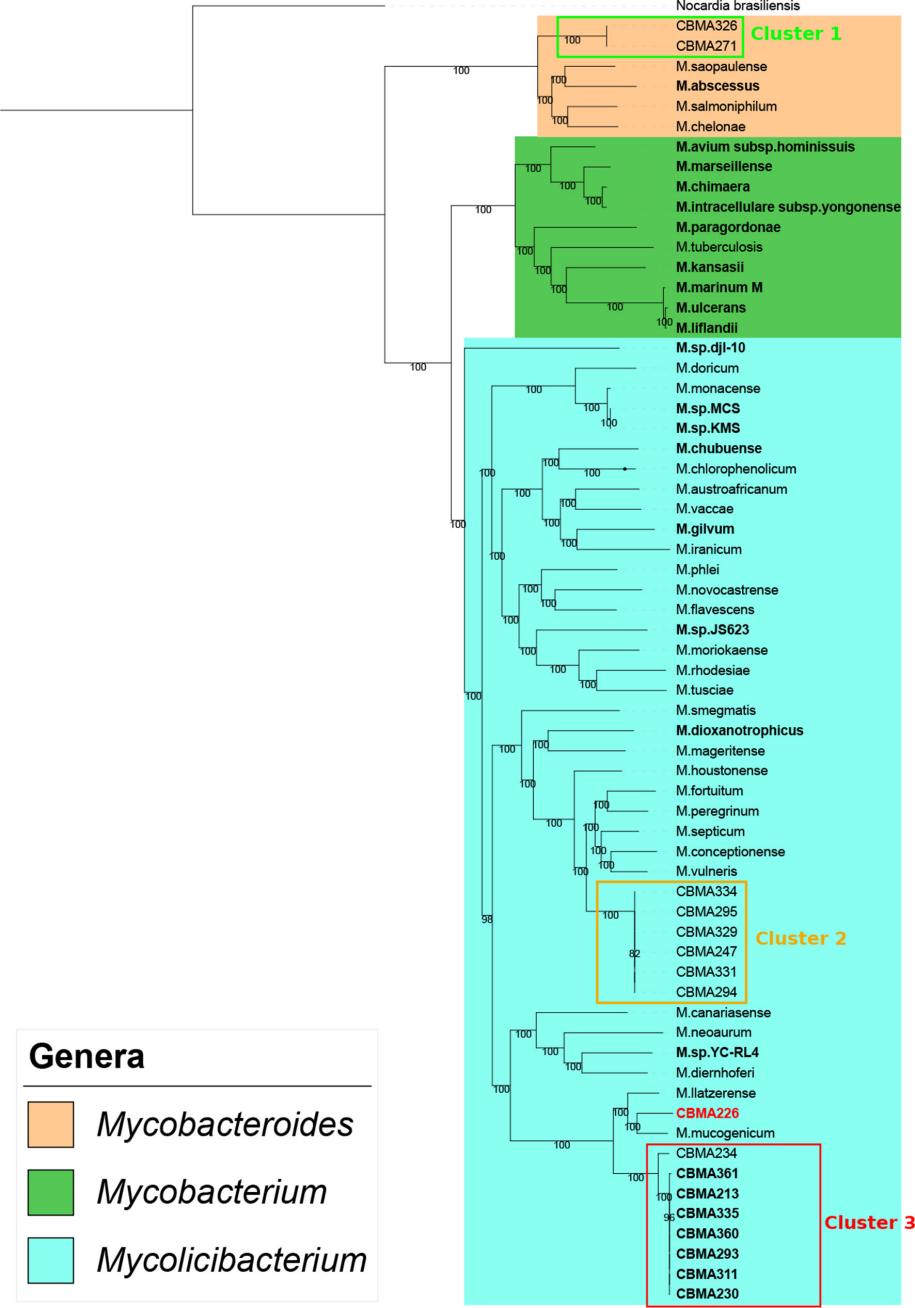
Maximum-likelihood core-genome tree based on 408 concatenated genes (totalling 338 kb). The different *Mycobacteriaceae* genera are depicted by the coloured backgrounds. Genomes harbouring plasmids are in bold. The CBMA genomes are delimited by the clusters, and the CBMA 226 is labelled in red.

Firstly, we performed a phylogenetic analysis to infer the genus of the *Mycobacteriaceae* CBMA strains. Based on the *Mycobacteriaceae* core genome, it was revealed that 15/17 CBMA strains belong to the *Mycolicibacterium* genus, while the other two, to the *Mycobacteroides* genus (Fig. 1). With the exception of *Mycolicibacterium* sp. CBMA 226, the CBMA strains grouped into three clusters, each associated with known *Mycobacteriaceae* species: cluster 1, *Mycobacteroides abscessus* complex species; cluster 2, *Mycolicibacterium fortuitum* complex species; and cluster 3, *Mycolicibacterium llatzerense* and *Mycolicibacterium mucogenicum* group. The CBMA genomes of cluster 1 and 2 corresponded to a single lineage in their respective groups, while cluster 3 presented two lineages composed by (i) *Mycolicibacterium* sp. CBMA 234 and (ii) *Mycolicibacterium* sp. CBMA 213, 230, 293, 311, 335, 360 and 361 (Fig. 1).

**Fig. 2.**
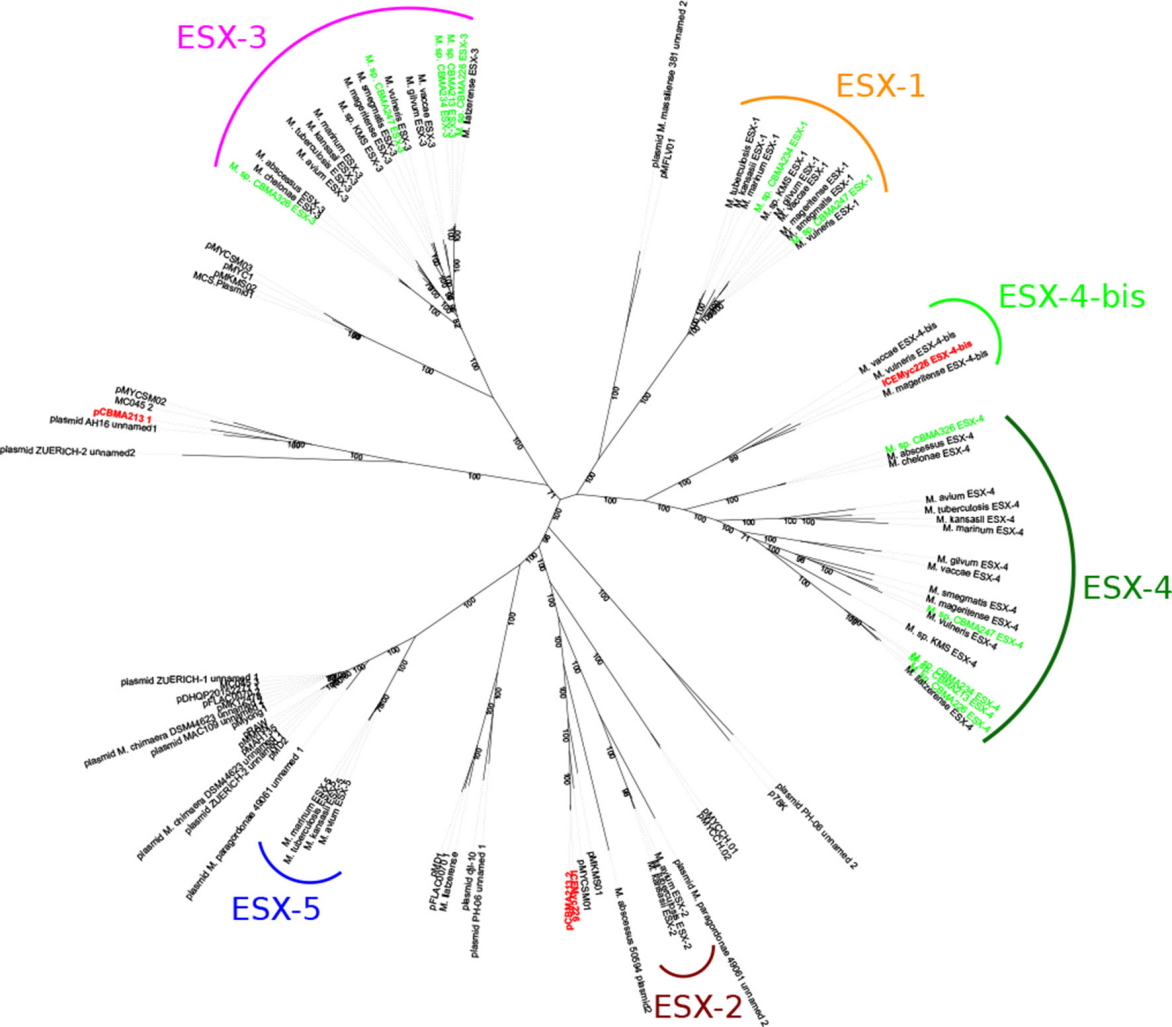
Maximum-likelihood tree of *Mycobacteriaceae* ESX loci based on concatenated T7SS core proteins. The coloured labels of the ESX types indicate the chromosome-borne ESX-systems, while others are plasmid-borne. The CBMA sequences are labelled in green (chromosome-borne) or red (mobile elements).

T7SS is a pivotal element in the survivor, communication and virulence of *Mycobacteriaceae *. Thus, we searched the genes resembling this apparatus in the CBMA genomes using hmm profiles and visual inspection. We identified clusters of ESX (T7SS) core genes in the chromosome of all CBMA strains. An ESX phylogeny was built using representatives of each CBMA lineage (* Mycolicibacterium* sp. CBMA 213, 226, 234, 247 and *Mycobacteroides* sp. CBMA 326), as well as ESX sequences identified in other *Mycobacteriaceae* species and plasmids (Fig. 2). ESX-3 and ESX-4 were identified in all CBMA chromosomes, while ESX-1 occurred only in *Mycolicibacterium* sp. CBMA 234 and CBMA 247 (Fig. 2). Another aspect observed in the ESX phylogeny was the separation of chromosome- and plasmid-borne ESX-systems, with the latter branching at the root of the former, indicating independent evolutionary processes.

### Mobilome identification

Using the search strategies, we identified three different plasmids (pCBMA213_1, pCBMA213_2 and pCBMA213_3) among genomes from cluster 3 (Fig. 1) and one integrative conjugative element (ICEMyc226) in *Mycolicibacterium* sp. CBMA 226 genome. Tables 1 and S3 summarize the major characteristics of each mobile element identified. Since plasmids were first identified in the *Mycolicibacterium* sp. CBMA 213, they were named referencing this genome and used as representative sequences in later analyses. All CBMA strains from cluster 3 (Fig. 1), except *Mycolicibacterium* sp. CBMA 234, presented at least one of the three plasmids in distinct combinations (Table 2). Each plasmid was identified as a single contig, except for pCBMA213_2 in *Mycolicibacterium* sp. CBMA 361, found fragmented into multiple contigs. In that case, we were unable to completely reconstruct the replicon. However, we observed that genes related to replication and mobility (*rep*A and relaxase) showed 100 % identity in comparison to the pCBMA213_2 sequences of the other CBMA genomes, suggesting the presence of pCBMA213_2 in *Mycolicibacterium* sp. CBMA 361. Although some plasmids are shared by these strains, they are not identical, since there is a small variation in their length (1–6 %) and number of genes (Table S4), which may be due to the genome assembly/sequencing procedure or natural variability. Some details of the different mobile elements are discussed below.

**Table 1.** Major features identified in the CBMA mobilome

Features	pCBMA213_1	pCBMA213_2	pCBMA213_3	ICEMyc226
Size (bp)	274 124	160 489	21 616	388 440
GC content	62 %	65 %	64 %	64 %
# CDS	328	161	27	364
Topology	Linear	Circular	Linear	Circular
*repA*		X	X	
relaxase		X		X
*virD4*		X		X
*virB4*	X	X		X
*tcpC*		X		X
Helicase	X			X
DNA polymerase	X			
Integrase	X			
Transposase	X	X		X
tRNA genes	X			
T7SS	X	X		X
Toxin-Antitoxin system	X	X		X

CDS, Coding DNA sequence.

**Table 2.** Plasmid distribution in the CBMA strains. The colours represent strains from the same phylogenetic group

	CBMA strains																
	**213**	**226**	**230**	**234**	**247**	**271**	**293**	**294**	**295**	**311**	**326**	**329**	**331**	**334**	**335**	**360**	**361**
pCBMA213_1	X														X		
pCBMA213_2	X						X								X	X	X*
pCBMA213_3	X		X				X			X					X	X	X

*Present in multiple contigs.

The pCBMA213_1 plasmid pCBMA213_1 (GenBank accession number MF600313.1) is a megaplasmid composed of 274 124 base pairs with an average GC content of 62 %, presenting a linear topology (Tables 1 and S3). Like other linear plasmids, pCBMA213_1 presented terminal inverted repeats, composed by 145 bp length with 100 % identity (Fig. S1a, b). blast analysis showed that pCBMA213_1 had no extended sequence similarity with other known plasmids, presenting best hit with the unnamed plasmid 1 in *Mycobacterium chimaera* AH16 strain (coverage of 17 % and identity of 67 %). Most of these shared sequences coded for hypothetical and T7SS genes. Indeed, the T7SS phylogeny of pCBMA213_1 showed that it clustered with the unnamed 1 plasmid of *M. chimaera* strain, branching at the root of the ESX-3 tree, despite belonging to a different genus, which suggests mobility of this ESX system (Fig. 2). Gene content analysis of pCBMA213_1 revealed sequences that seem to be involved in DNA replication, such as a ribonuclease HI gene (B5P44_p00084), and a 10 kb region (11 to 21 kb) encoding DNA polymerase I (B5P44_p00015), DNA segregation ATPase (FtsK/SpoIIIE family)(B5P44_p00018), and ParA (B5P44_p00022) proteins. Although we could not find a *rep* gene, pCBMA213_1 also encoded a replicative DNA helicase (DnaB-like) (B5P44_p00269) with high similarity to helicase sequences from *Mycolicibacterium* sp. JS623 plasmid pMYCSM03 (94 % coverage and 75 % identity) and *Mycobacterium chimaera* strain ZUERICH-2 plasmid unnamed 2 (89 % coverage and 72 % identity). Interestingly, a ~89 kb region (169 352–258 453 bp), which encodes the 32 tRNA genes, is flanked by transposase genes with 90 % identity (B5P44_p00196 and B5P44_p00348) and inverted repeats (101 bp length with 99 % identity) (Fig. S1c, d). These tRNA genes were explored in a previous study [58]. Moreover, pCBMA213_1 also carries three Toxin-Antitoxin (TA) systems of different type-II TA families (Table S5).

The pCBMA213_2 plasmid pCBMA213_2 (GenBank accession number KY349138.1) is another megaplasmid, composed of 160 489 base pairs with an average GC content of 65 %, and unlike pCBMA213_1, its topology is circular. This plasmid is characterized by the presence of genes related to replication, mobility and conjugation, such as *rep*A, relaxase, transposase, T4SS-like and T7SS genes (Tables 1 and S3). In addition, pCBMA213_2 also carries one TA system (Table S5). blast analysis showed similarity of the pCBMA213_2 sequence with *Mycolicibacterium* sp. JS623 plasmid pMYCSM01 (21 % coverage and 71 % identity) and *Mycolicibacterium* sp. KMS plasmid pMKMS01 (22 % coverage and 67 % identity). Like the pCBMA213_1 plasmid, the similarity of pCBMA213_2 with other plasmids is mainly due to the T7SS sequences. This can be seen in the T7SS phylogeny, where pCBMA213_2 branched at the root of the ESX-2 tree (Fig. 2). The analysis of pCBMA213_2 ESX system was presented in a previous study [29].

The pCBMA213_3 plasmid pCBMA213_3 (GenBank accession number MN587875) is the smallest plasmid found in the CBMA genomes, composed of 21 616 base pairs with an average GC content of 64 % (Table 1). Its topology is linear, with terminal inverted repeats of 489 bp length with 100 % identity (Fig. S2). pCBMA213_3 appears to be a cryptic plasmid capable of autonomous replication, since almost all predicted CDS were hypothetical, except for a putative *rep* gene (pCBMA213_3_00008). blast analysis showed that CBMA213_3 shows similarity to the sequences (encoding hypothetical genes) of two *Mycobacterium* plasmids: *Mycobacterium* sp. MOTT plasmid pM90 (17 % coverage and 68 % identity) and *Mycobacterium* celatum plasmid pCLP (19 % coverage and 69 % identity). These plasmids share similar characteristics with pCBMA213_3, since they are small (18 and 23 kb length), and plasmid pCLP has linear topology [20]. Curiously, pCBMA213_3 shares a gene encoding a DUF4189 domain-containing protein with pCBMA213_1 (respectively, pCBMA213_3_00027 and B5P44_p00076).

### Characterization of an integrative conjugative element

**Fig. 3.**
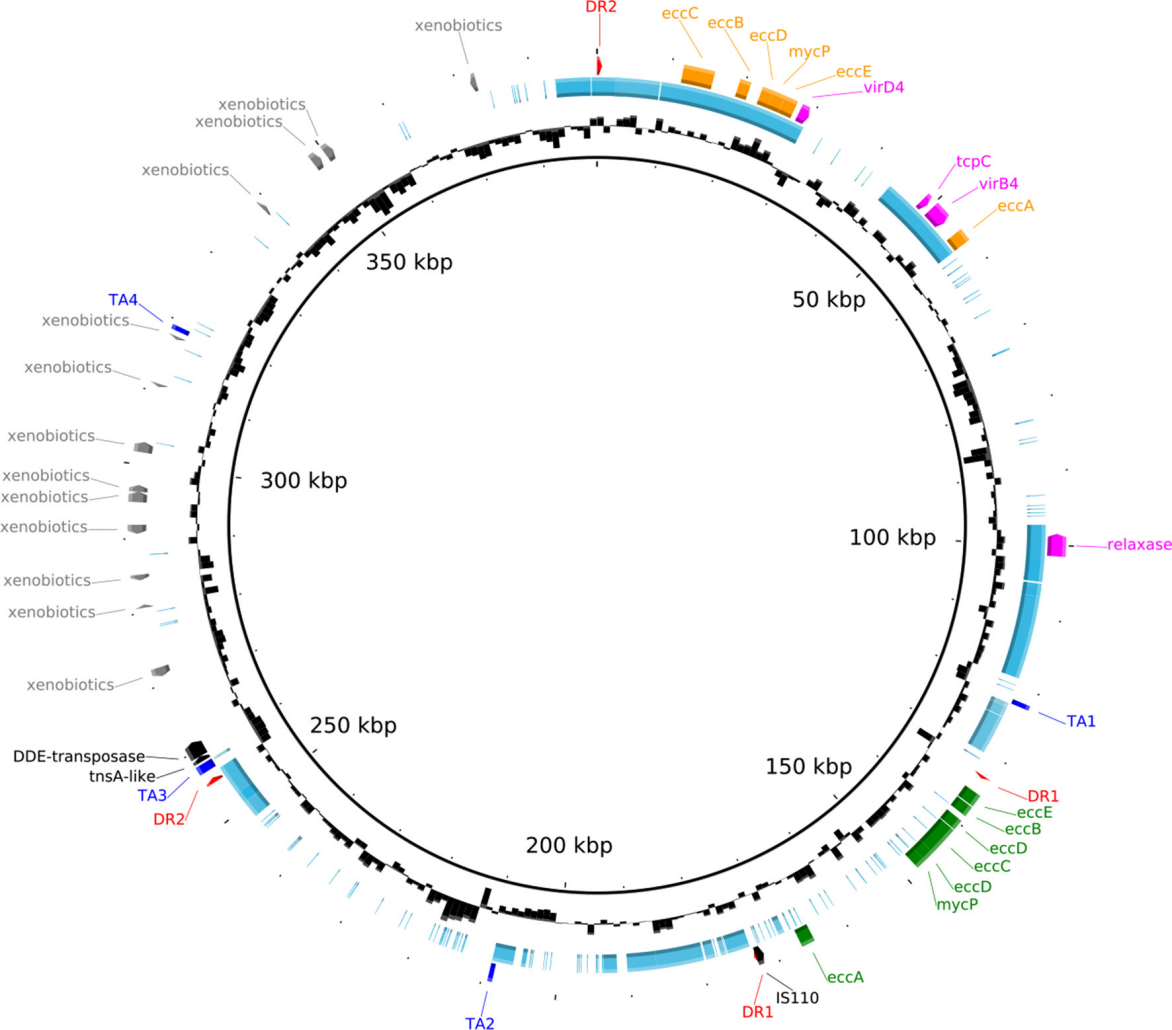
Genomic map of ICEMyc226 highlighting major features and segments shared with pCBMA213_2 (light blue segments). The external coloured blocks represent the regions that encode for: Toxin-Antitoxin systems (dark blue: TA1, TA2, TA3, and TA4); each end of the direct repeats (red: DR1 and DR2); xenobiotic degrading genes (grey: xenobiotics); transposase elements (black: DDE-transposase, tnsA-like, and IS110); ESX-2-like genes (orange: eccC, eccB, eccD, mycP, eccE and eccA); T4SS-like genes (fuchsia: virD4, tcpC, virB4 and relaxase); ESX-4-bis genes (green: eccE, eccB, eccD, eccC, eccD, mycP, eccA). The second circle, from the inside out, represents the GC content of ICEMyc226.

Analysing the *Mycolicibacterium* sp. CBMA 226 genome, we observed a single contig encoding a set of genes that characterize ICEs, including a relaxase (ICEMyc226_00083), T4SS-like genes (ICEMyc226_00024, ICEMyc226_00036, ICEMyc226_00039) and a DDE-type integrase/transposase/recombinase with the Rve protein domain of retrovirus integrases (ICEMyc226_00243; Tables 1 and S3). ICEMyc226 (GenBank accession number MN587876) is composed of 388 440 base pairs with an average GC content of 64 %, and encodes 364 CDS (Table 1). It was predicted as a circular molecule (Fig. 3), characterizing it as an ICE in its excised form. This element encoded a large number of genes related to partition and maintenance systems, including five genes that encode ParA/B-like family proteins, and four type II TA systems (Table S5). blastp analyses, using all predicted proteins encoded by ICEMyc226 (*n*=364), against the ICEberg database showed that 101 proteins showed similarities with other ICE proteins, including transposases, VirD4, metabolic and regulatory proteins (22–52 % identity). Interestingly, ICEMyc226 DDE-type integrase/transposase/recombinase showed similarity (~30 % identity) with an integrase of TR2 ICE (154 kb length) from *Streptomyces scabiei* 87.22, and of other ICEs from *Proteobacteria* (Fig. S3). No antibiotic resistance genes were identified in ICEMyc226.

ICEMyc226 encoded two ESX-systems, one branched at the root of the ESX-2 tree with other plasmid-borne ESX-systems, and the other clustered on ESX-4-bis with chromosome-borne ESX-systems (Fig. 2). When the ESX-4-bis sequences from ICEMyc226 and *Mycolicibacterium mageritense* were compared, the same ESX gene order was observed: *ecc*E/*ecc*B/*ecc*D/*ecc*C/*ecc*D/*myc*P/-//-/*ecc*A (*ecc*A located ~17 kb downstream). Interestingly, the ESX-4-bis region of ICEMyc226 (~40 kb) is flanked by direct repeats of 167 bp with 100 % identity (Fig. 3 - DR1), and an IS110 transposase gene is found downstream this ESX locus, adjacent to the direct repeat (Fig. 3). The other ESX locus of ICEMyc226, ESX-2-like, is similar to the ESX of pCBMA213_2 (99 % coverage and 92 % identity). The synteny and identity of the genes of this ESX locus in both elements are conserved in relation to the ESX locus of *Mycolicibacterium* sp. KMS pMKMS01 plasmid: *ecc*C/*ecc*B/*ecc*D/*myc*P/*ecc*E/-//-/*ecc*A (*ecc*A downstream) (Fig. 2). In both ICEMyc226 and pCBMA213_2, the region between *ecc*E and *ecc*A encodes several genes, including the T4SS-like genes: *vir*B4, *vir*D4 and *tcp*C (*vir*B8-like). This region is larger in ICEMyc226 due to an ~14 kb insertion, which encodes two PE-PGRS family protein PE_PGRS18, two-component LuxR family transcriptional regulator, an ATP-binding protein, and a hypothetical protein (Fig. 3).

Since pCBMA213_2 and ICEMyc226 shared a large syntenic block of genes, we performed a comparative analysis of them. pCBMA213_2 is 160 kb in length, and ~70 % of its content (~112 kb) is shared with segments of ICEMyc226 with ~90 % identity, which corresponds to ~30 % of the ICE size. The shared segments include genes associated with the conjugative machinery (T7SS, T4SS-like, and relaxase genes), flanked by direct repeats of 648 bp with 100 % identity (Fig. 3 - DR2) and adjacent to the DDE-type integrase/transposase/recombinase (Fig. 3). Notably, ICEMyc226 lacks the *rep*A gene and 53 mostly hypothetical coding sequences that are present in pCBMA213_2.

**Fig. 4.**
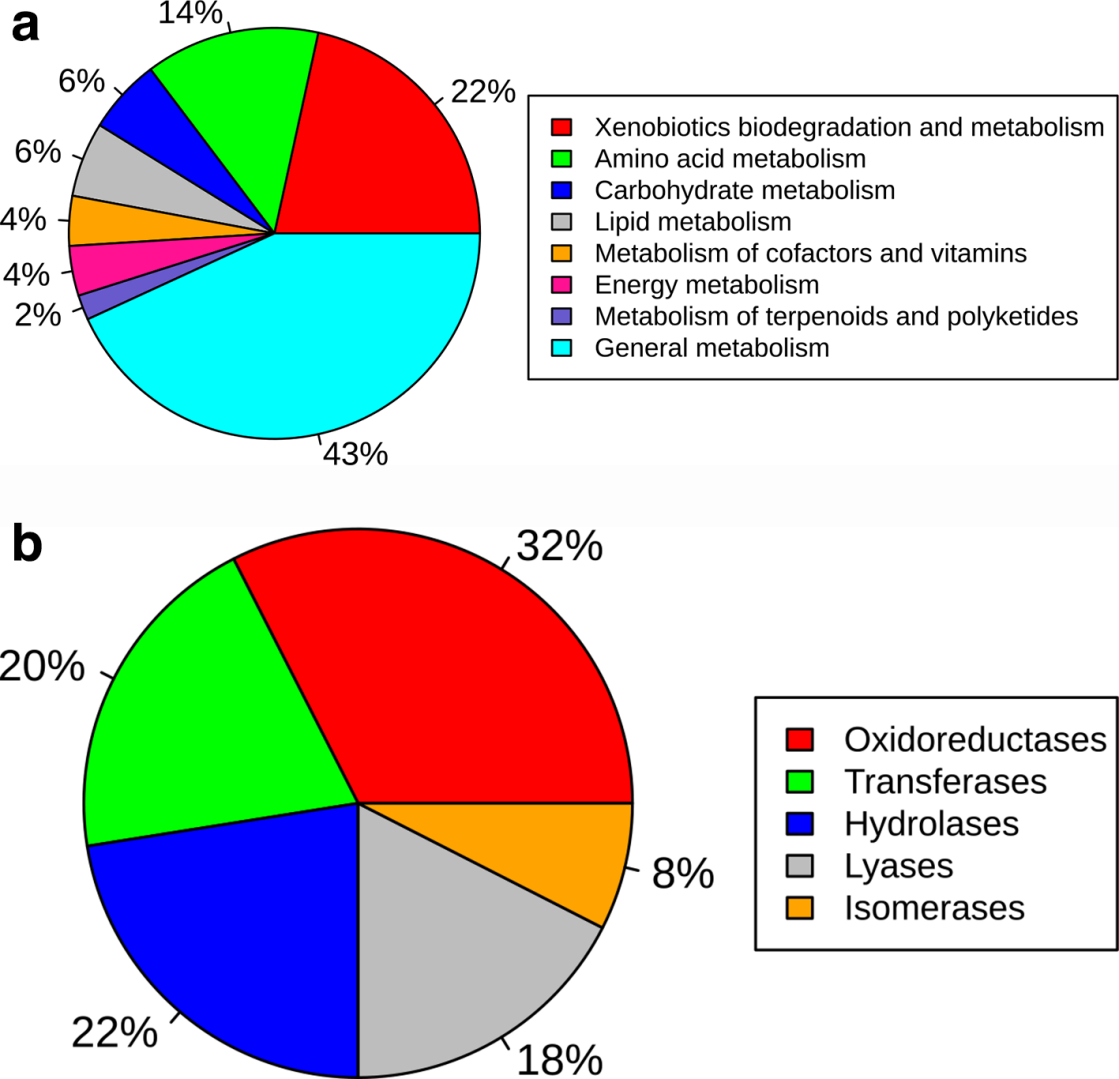
Distribution of KEGG metabolic categories among ICEMyc226 CDS, including metabolic pathways (a) and enzymatic functions (b).

Gene content analysis of ICEMyc226 revealed that ~21 % of the coding sequences (78 CDS) presented similarity with *Actinobacteria* and *Proteobacteria* sequences*,* raising evidence that some genes were horizontally acquired. Moreover, Alien_Hunter software predicted that ~131 kb out of 388 kb (ICEMyc226 length) are related to putative HGT events (Table S6). The functional inference of the ICEMyc226 genome revealed 55 genes related to metabolic processes, such as metabolism of xenobiotics (Fig. 4a) and enzymatic functions (Fig. 4b). Most of the genes associated with xenobiotics metabolism were involved with benzoate (*n*=4) and styrene (*n*=3) degradation. A global overview of the metabolic pathways associated with ICEMyc226 genes allowed us to determine the range of processes that this element can influence (Fig. S4). Although ICEMyc226 lacks complete sets of genes needed to metabolize molecules independently of the host, depending on the environment, it could still metabolically complement its host.

## Discussion

The *Mycobacteriaceae* mobilome has been extensively explored in relation to bacteriophages [14]. However, to date, there is a lack of information regarding other mobile elements, such as plasmids and genomic islands. Even so, plasmids had a pivotal role in the evolution of this family, as they were involved in the diversification and mobility of T7SS [31–33]. Here, we explored the mobilome of *Mycobacteriaceae* strains from the soil of the Brazilian Atlantic Forest, which is one of the main biodiversity hotspots in the world and has a prevalence of *Mycobacteriaceae* [59, 60]. Interestingly, we identified a T4SS-mediated ICE, together with circular and linear plasmids in lineages from the *Mycolicibacterium* genus. These mobilome elements were restricted to only some of the lineages analysed (Fig. 1, Table 2). So far, only a few AICEs had been reported in genomes of bacteria from this genus [9, 34]. ICEMyc226, like ICEs from other bacterial families, has a backbone of characteristic genes [2, 3], besides two ESX-systems and a set of xenobiotic degrading genes. Interestingly, this is the first evidence of ESX-systems in other mobile elements, besides plasmids.

The CBMA plasmids are distinct from each other, showing differences in size, topology, replication system, conjugative trait, etc. The linear CBMA plasmids, pCBMA213_1 and pCBMA213_3, like that of other linear mycobacteria plasmids, had inverted terminal repeats [18, 25]. However, pCBMA213_1 seems to have a distinct replication system since it does not carry a *rep* gene [61]. Instead, pCBMA213_1 encoded a DnaB-like replicative helicase, which have been reported to act in the replication of *Streptomyces* linear plasmids and would characterize this plasmid as a replicon [62, 63]. Interestingly, despite its linear topology, pCBMA213_1 encodes colE1-like elements of theta-type circular plasmids, such as DNA polymerase and RNAse H [64, 65]. A relatively uncommon feature, namely a tRNA array (32 tRNA genes clustered in ~12 kb), had been previously characterized in this replicon [58]. In the case of the circular pCBMA213_2 replicon, it was possible to infer its conjugative nature due to the presence of the relaxase, T4SS and T7SS genes. This set of genes has been shown to play a role in *Mycobacterium* plasmid conjugation [27]. T7SS is found at one copy in several *Actinobacteria* plasmids [27, 28, 30–33]. In fact, pCBMA213_1 and pCBMA213_2 encoded ESX-3-like and ESX-2-like, respectively. These two ESX-systems carried by the CBMA plasmids have a close phylogenetic relationship with the ESX-systems of other distinct plasmids that are carried by several species from different niches (Fig. 2). The ESX-3 system is related to metal homeostasis, while the ESX-2 function is still unknown [33]. In addition to the chromosomally encoded ESX-3 and ESX-4 systems, the host strain has an extra ESX-3 copy, which would impact the bacterial fitness in the environment. These results reinforce the hypothesis of the mobilization of the ESX-system through mobile genetic elements, which have driven its evolution and mobility [31–33].

ICEMyc226 encodes an ESX-2-like system that is similar to the one encoded by pCBMA213_2 (Fig. 2). Interestingly, these elements belong to strains from different species (Fig. 1), indicating a horizontal gene transfer event. Both ICE and plasmid also share relaxase and T4SS genes with high identities (~90 %) which, in association with the ESX-2-like system, represent the ICEMyc226 conjugative module. Therefore, we hypothesized an ancestral fusion between pCBMA213_2 and an integrative mobilizable element (IME; e.g. genomic island), with subsequent recombination events, resulting in the current ICEMyc226. IMEs encode genes related to integration and excision and may hijack or subvert the mating apparatus of conjugative elements to promote their own transfer [3, 66–68]. The recombination of some mobile elements, including IMEs and ICEs, rely on DDE-transposases instead of integrases, and this type of enzyme may be associated with the conjugative apparatus of the mobile elements [2, 3, 10, 69–73]. Indeed, plasmids can become ICEs by acquiring integrases from other mobile elements [2, 74], and recombination among mobile elements seems to be frequent during the evolution of plasmids and ICEs [7, 75, 76]. Curiously, DDE-transposases are widespread in Gram-positive bacteria [77].

In addition to carrying the ESX-2-like, the ICEMyc226 also carries an ESX-4-bis. This finding is unique considering the mobile genetic elements of *Mycobacteriaceae* that harbour ESX-systems, since only one ESX-system was observed in plasmids. The ICEMyc226 ESX-4-bis has a close phylogenetic relationship with chromosome-borne ESX-4-bis of other strains (Fig. 2). This suggests a recent transmission event, probably mediated by IS110, within *Mycobacteriaceae *, which corroborates the transmissible character of T7SS among replicons [31–33].

*Proteobacteria* ICEs often carry accessory genes related to antibiotic resistance that impact the clinic [78, 79]. Here, we did not identify antibiotic resistance genes in ICEMyc226; instead, there is a prevalence of genes related to xenobiotics and amino acid metabolism. Indeed, ICEs are more likely to encode metabolism-related genes than antibiotic resistance genes [74]. Moreover, dozens of ICEMyc226 genes presented high identity with genes from non-* Mycobacteriaceae* bacteria, revealing the occurrence of HGT events between ICEMyc226 host and other soil bacteria genera. Indeed, HGT events have already been observed between *Mycobacteriaceae* and other genera of *Actinobacteria* and *Proteobacteria* [80].

Altogether, this study uncovers an aspect of the underexplored diversity of *Mycobacteriaceae* mobilome, showing evidence of unique plasmids and an ICE in its repertoire. In particular, the ICE appears to have been the result of a recombination between one of the identified plasmids and another mobile element. Also, this ICE encodes two ESX-systems, one of which presents evidence of mobilization. Thus, besides plasmids, other mobile genetic elements, such as ICEs, could have participated in the spread of T7SS in *Mycobacteriaceae *. The results presented here reiterate the need for studies with environmental samples to unravel the mobilome diversity of these organisms.

## Data Bibliography

1. Raw reads have been deposited at the NCBI under the Bioproject number PRJNA344484.

2. Accession numbers of publicly available genomes used for the core genome multilocus sequence analysis are reported in Table S1.

3. Accession numbers of publicly available plasmids used for the comparative analysis are reported in Table S2.

## Supplementary Data

Supplementary material 1

Supplementary material 2
