# Supplementary material for: Genomics of Atlantic Forest Mycobacteriaceae strains unravels a mobilome diversity with a novel integrative conjugative element and plasmids harbouring T7SS
Source: Microb Genom. 2020 Jun 4;6(7):mgen000382. doi: 10.1099/mgen.0.000382 (PMC7478629; doi:10.1099/mgen.0.000382)
Supplement: Supplementary material 1 [file mgen-6-382-s001.pdf]

Supplementary Figures

Figure S1

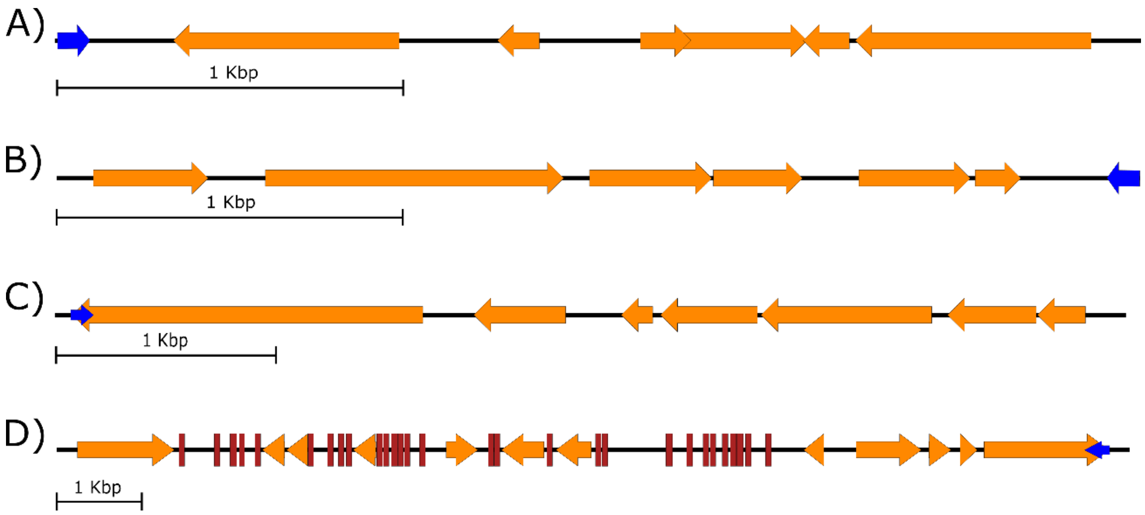

Fig. S1. Inverted repeats of pCBMA213_1. Inverted terminal repeats of pCBMA213_1 on the left side (A) and on right side (B). Inverted repeats flanking the left (C) and right (D) sides of a ~89 kb region (169,352 - 258,453 bp). The blue and orange arrows represent the inverted repeats and protein-coding genes, respectively, while the red blocks represent tRNA genes. The scale is shown below each figure.

Figure S2

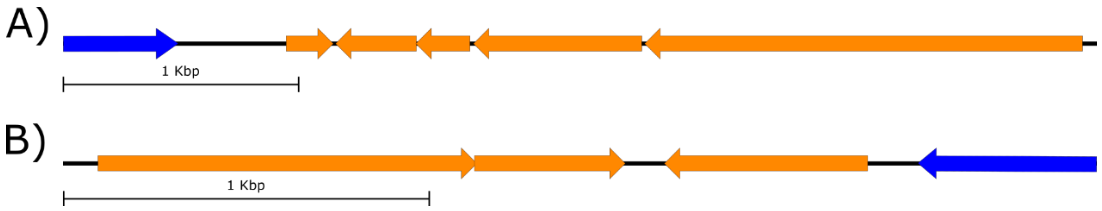

Fig. S2. Inverted terminal repeats of pCBMA213_3 on the left side (A) and on right side (B). The blue and orange arrows represent the inverted terminal repeats and protein-coding genes, respectively. The scale is shown below each figure.

Figure S3

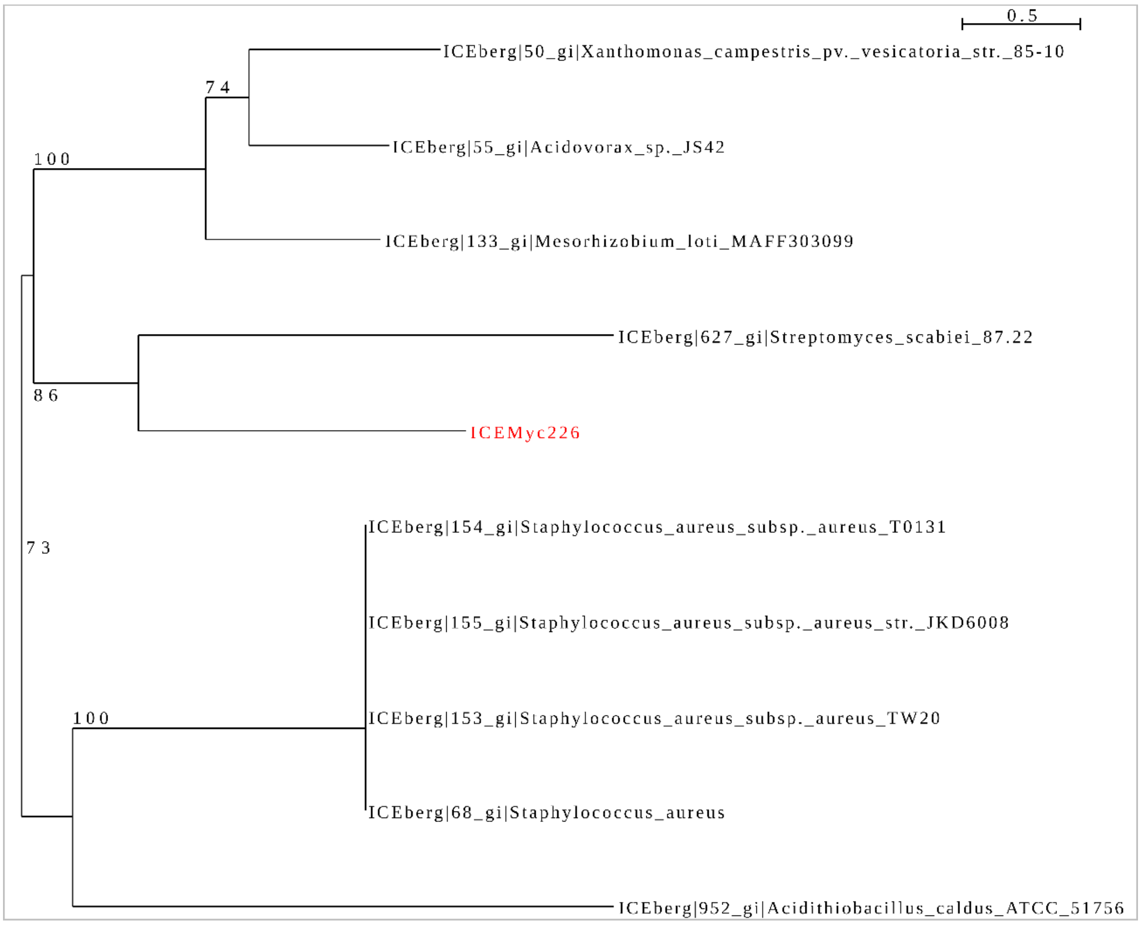

Fig. S3. Maximum likelihood tree (100 bootstrap replicates) of ICEMyc226 DDE-integrase/transposase/recombinase with the most similar integrases and transposases from the ICEberg database.

Figure S4

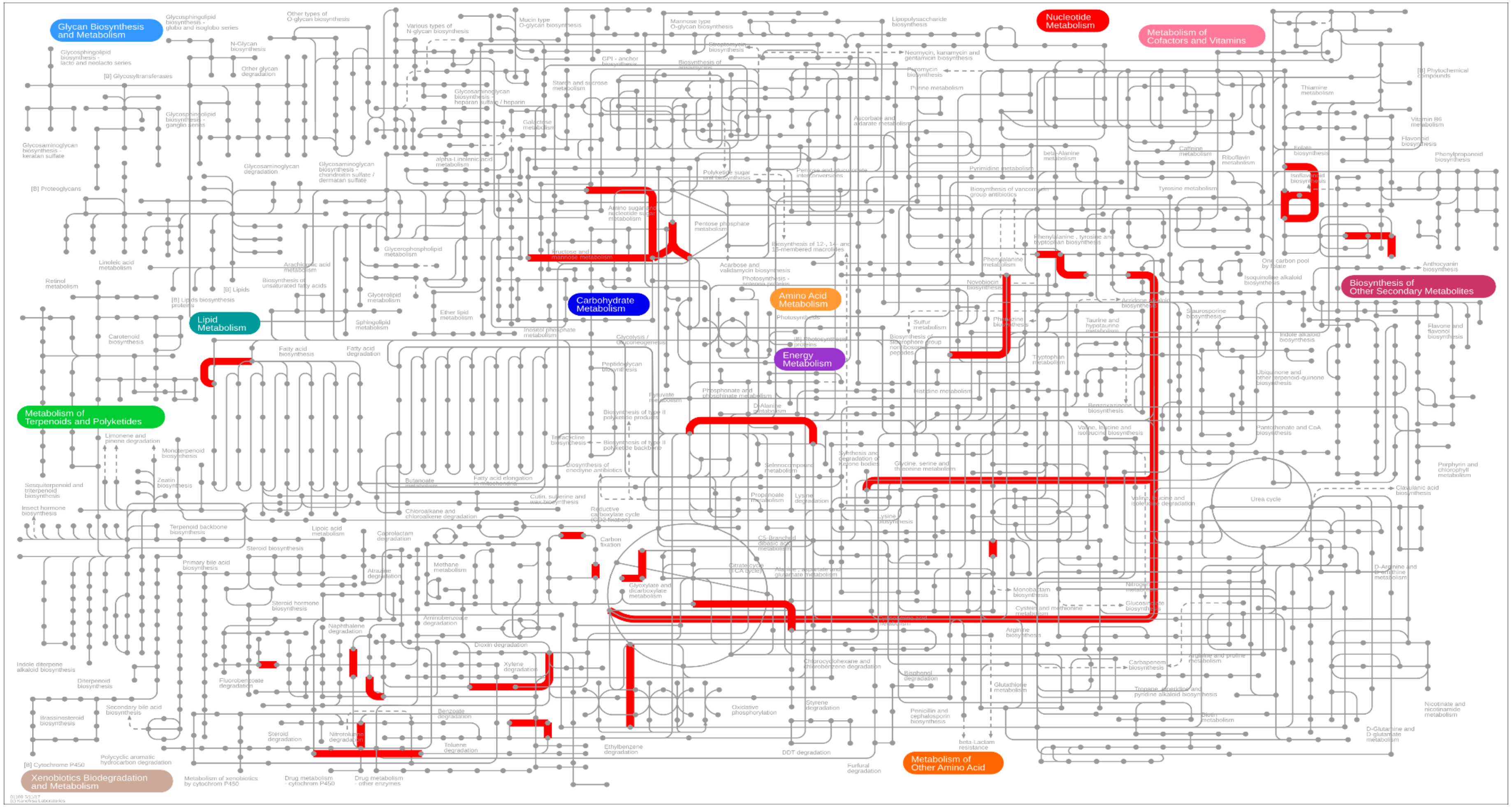

Fig. S4. Mapping of ICEMyc226 genes onto the KEGG metabolic pathways. ICEMyc226 genes associated with metabolic pathways (red thick lines) were mapped following the KEGG Orthology (KO).
